# Recovery in Schizophrenia: The Role of Psychosocial interventions

**DOI:** 10.1192/j.eurpsy.2024.65

**Published:** 2024-08-27

**Authors:** T. Wykes

**Affiliations:** Psychology, King’s College London, London, United Kingdom

## Abstract

**Abstract:**

**Recovery in Schizophrenia: The Role of Psychosocial interventions** Recovery is individual and so needs individual responses from the mental health services. Different interventions are useful at different stages and of course they only “work” for some people. The paper will describe some psychosocial interventions and the role they might pay in the patient’s journey to their expected recovery. Three main strategies are often referred to – reducing symptoms, reducing barriers to recovery, and extending and maintaining recovery to achieve some stable and acceptable (to the patient) optimal level of functioning. Psychosocial intervention strategies are beneficial for each of these often thought of as independent, but they are inter-related with one type of therapy leading to reductions in the need for other therapies. The process of considering which one to start with is a choice and this paper will describe some decision making to ensure that patients have the best options.

**Disclosure of Interest:**

None Declared

